# Supplementary material for: Embodied Semantics: Early Simultaneous Motor Grounding in First and Second Languages
Source: Brain Sci. 2024 Oct 25;14(11):1056. doi: 10.3390/brainsci14111056 (PMC11591616; doi:10.3390/brainsci14111056)
Supplement: Supplementary file 1 [file brainsci-14-01056-s001.zip › brainsci-3227451-supplementary.pdf]

List of the 200 motor and non motor stimuli used during the EEG task;
M = motor; NM = Non Motor

GERMAN STIMULI	bloc	verbtype	FRENCH STIMULI	bloc	verbtype
nehmen	1	M	prendre	2	M
bügeln	2	M	repasser	1	M
greifen	1	M	saisir	2	M
unterschreiben	2	M	signer	1	M
zuschneiden	1	M	tailler	2	M
stempeln	2	M	tamponner	1	M
steuern	1	M	conduire	2	M
drücken	2	M	appuyer	1	M
zeichnen	1	M	dessiner	2	M
einpacken	2	M	emballer	1	M
wegnehmen	1	M	enlever	2	M
befestigen	2	M	attacher	1	M
schminken	1	M	maquiller	2	M
mischen	2	M	mélanger	1	M
zeigen	1	M	montrer	2	M
kämmen	2	M	peigner	1	M
aufschneiden	2	M	insérer	2	M
wegstellen	1	M	chiffonner	1	M
aufhängen	2	M	saler	2	M
schneiden	1	M	beurrer	1	M
zerreissen	2	M	tartiner	2	M
packen	1	M	vacciner	1	M
schiessen	2	M	sculpter	2	M
schneidern	1	M	trier	1	M
schnitzen	2	M	bricoler	2	M
streichen	1	M	agiter	1	M
vergraben	2	M	pincer	2	M
schieben	1	M	verser	1	M
klatschen	2	M	pointer	2	M
winken	1	M	cuisiner	1	M
ziehen	2	M	serrer	2	M
anfassen	1	M	ranger	1	M
putzen	2	M	couvrir	2	M
umarmen	1	M	nettoyer	1	M
zuschliessen	2	M	laver	2	M
knüpfen	1	M	attraper	1	M
falten	2	M	fermer	2	M
aufsammeln	1	M	offrir	1	M
sticken	2	M	ouvrir	2	M
werfen	1	M	tirer	1	M
hacken	1	M	tambouriner	2	M
klopfen	2	M	colorier	1	M
nähen	1	M	feuilleter	2	M

nageln	2 M	tricoter	1 M
schreiben	1 M	brosser	2 M
malen	2 M	balayer	1 M
hämmern	1 M	découper	2 M
berühren	2 M	détacher	1 M
graben	1 M	arroser	2 M
schlagen	2 M	cueillir	1 M
zuteilen	2 NM	allouer	1 NM
verbessern	1 NM	améliorer	2 NM
kennen	2 NM	connaître	1 NM
bestehen	1 NM	consister	2 NM
raten	2 NM	deviner	1 NM
zweifeln	1 NM	douter	2 NM
urteilen	2 NM	juger	1 NM
denken	1 NM	penser	2 NM
vorgeben	2 NM	prétendre	1 NM
vorsehen	1 NM	prévoir	2 NM
vorschlagen	2 NM	proposer	1 NM
vermuten	1 NM	supposer	2 NM
übersetzen	2 NM	traduire	1 NM
untersagen	1 NM	consentir	2 NM
entwerfen	2 NM	mémoriser	1 NM
bewundern	1 NM	attribuer	2 NM
erteilen	2 NM	acquérir	1 NM
befolgen	1 NM	réessayer	2 NM
lehren	2 NM	prédire	1 NM
rechnen	1 NM	oser	2 NM
fürchten	2 NM	raisonner	1 NM
zählen	1 NM	assumer	2 NM
scheinen	2 NM	soumettre	1 NM
mögen	1 NM	sembler	2 NM
ändern	2 NM	conseiller	1 NM
heissen	1 NM	céder	2 NM
tagträumen	2 NM	fournir	1 NM
versteuern	1 NM	promettre	2 NM
abschaffen	2 NM	apprendre	1 NM
erwägen	1 NM	délibérer	2 NM
fördern	2 NM	envier	1 NM
verachten	1 NM	débuter	2 NM
verbieten	2 NM	déduire	1 NM
erben	1 NM	estimer	2 NM
langweilen	2 NM	exagérer	1 NM
versichern	1 NM	distinguer	2 NM
bedauern	2 NM	décevoir	1 NM
aussuchen	1 NM	transmettre	2 NM
ärgern	2 NM	accorder	1 NM
täuschen	1 NM	adorer	2 NM
besitzen	2 NM	négocier	1 NM

fehlen	1 NM	obéir	2 NM
wählen	2 NM	rêver	1 NM
hassen	1 NM	rater	2 NM
hoffen	2 NM	reconnaître	1 NM
wünschen	1 NM	permettre	2 NM
glauben	2 NM	surveiller	1 NM
wollen	1 NM	réfléchir	2 NM
wissen	2 NM	oublier	1 NM
einfügen	1 NM	perdre	2 NM
